# Efficacy and pharmacoeconomic advantages of Fufang Huangbai Fluid hydropathic compress in diabetic foot infections: a comparative clinical study with antimicrobial calcium alginate wound dressing

**DOI:** 10.3389/fphar.2024.1285946

**Published:** 2024-01-22

**Authors:** Guangyao Yang, Gang Wang, Zhenghong Li, Lijuan Deng, Ning Wang, Xuewan Wang, Tong Zhou, Jingming Zhang, Yin Lei, Tao Wang, Yue Wang, Hanying Shao, Mingya Chen, Keren Zhang, Min Zhou, Xiangbao Wang, Xingfang Liu, Shang Ju

**Affiliations:** ^1^ Beijing Hepingli Hospital, Beijing, China; ^2^ Beijing University of Chinese Medicine, Beijing, China; ^3^ Department of Peripheral Vascular, Dongzhimen Hospital, Beijing University of Chinese Medicine, Beijing, China; ^4^ Research Department, Swiss University of Traditional Chinese Medicine, Bad Zurzach, Switzerland; ^5^ Department of Interventional Center, Dongzhimen Hospital, Beijing University of Chinese Medicine, Beijing, China

**Keywords:** Fufang Huangbai Fluid (FFHB), antimicrobial efficacy, clinical trial, Chinese medicine, pharmacoeconomics, diabetes

## Abstract

**Objective:** To compare the intervention effects and pharmacoeconomic advantages of Fufang Huangbai Fluid (FFHB) hydropathic compress *versus* Antimicrobial Calcium Alginate Wound Dressing (ACAWD) in the treatment of diabetic foot infections (DFI).

**Methods:** Patients with DF who were hospitalized in the peripheral vascular Department of Dongzhimen Hospital of Beijing University of Chinese Medicine from December 2020 to February 2022 and met the inclusion and excluding criteria were allocated into the experimental group and control group through minimization randomization. The experimental group was treated with FFHB hydropathic compress for 2 weeks, while the control group was treated with ACAWD for the same duration. The wound healing of both groups was monitored for 1 month post-discharge. Clinical data from all eligible patients were collected, and differences in various indices between cohorts were analyzed.

**Results:** 22 in the experimental group (including two fell off) and 20 in the control group. After the treatment, the negative rate of wound culture in the experimental group was 30% and that in the control group was 10%, There was no significant difference in the negative rate of wound culture and change trend of minimum inhibitory concentration (MIC) value of drug sensitivity (*p* > 0.05). The infection control rate of the experimental group was 60%, and that of the control group was 25%. The difference between the two groups was statistically significant (χ2 = 5.013, *p* = 0.025). The median wound healing rate of the experimental group was 34.4% and that of the control group was 33.3%. There was no significant difference between the two groups (*p* > 0.05). During the follow-up 1 month later, the wound healing rate in the experimental group was higher, and the difference was statistically significant (*p* = 0.047). Pharmacoeconomic evaluations indicated that the experimental group had greater cost-effectiveness compared to the control group.

**Conclusion:** In the preliminary study, FFHB demonstrated comparable pathogenic and clinical efficacy to ACAWD in the treatment of mild DF infection, and exhibited superior pharmacoeconomic advantages. With the aid of infection control, the wound healing rate in the FFHB group showed notable improvement. Nevertheless, due to the limited sample size, larger-scale studies are warranted to further validate these findings.

**Clinical Trial Registration**: (https://www.chictr.org.cn/showproj.aspx?proj=66175), identifier (ChiCTR2000041443).

## 1 Introduction

Diabetic foot infection (DFI) is not only a significant factor leading to the deterioration of diabetic foot (DF), but it also stands as the most prevalent cause of hospitalization and even amputation in DF patients (Lavery et al., 2007; Ndosi et al., 2018; Tan et al., 2019). A prospective study reported that DFI patients had only a 46% healing rate within 1 year (another 10% of these patients would relapse), a mortality rate of 15%, and an amputation rate of 17% (Tan et al., 2019). If patients become infected, they face a higher likelihood of amputation. Studies by Lavery et al. have shown that the risk of amputation in DF patients with lower limb infection is 154.5 times higher than that in patients without infection (Lavery et al., 2006). A meta -analysis examining risk factors for large amputation in DF patients showed that major amputation in DF patients was associated with infection (OR:2.5295%CI:1.71–3.71) (Wang et al., 2018). The presence of infection makes DFI wounds challenging to heal, which seriously affects the quality of life of patients, occupies a lot of medical resources, and brings a heavy burden to the family and society.

Fufang Huangbai Fluid (FFHB) Comprises five traditional Chinese medicine: Forsythiae Fructus [Oleaceae; Forsythia suspensa fruit], Phellodendri Chinensis Cortex [Rutaceae; Phellodendron amurense bark], Lonicerae Japonicae Flos [Caprifoliaceae; *Lonicera japonica* Thunb flower bud], Taraxaci Herba [Compositae; Taraxacum mongolicum Herb], Scolopendra [Scolopendridae; Scolopendra subspinipes Mutilans whole worm]. The plant names were verified at http://mpns.kew.org/mpns-portal, and the name of Scolopendra was authenticated using the Pharmacopoeia of the People’s Republic of China. FFHB is believed to clearing away heat and toxic materials, reducing swelling, and eliminating decay. Studies have shown that the effective rate of treating DF wounds infected with methicillin-resistant *Staphylococcus aureus* with FFHB is 92% (Wang et al., 2019).

Currently, most clinical studies focus primarily on clinical observation to assess FFHB’s therapeutic effects, and the research on the effect of FFHB on pathogenic microorganisms of DFI wound is limited to basic medical research. At present, there is still a lack of evaluation on the intervention effect of FFHB on the wound pathogenic microorganisms of real DFI patients.

## 2 Materials and methods

### 2.1 Design and participants

Forty-two patients with DF, admitted to the Department of Peripheral Vascular at Dongzhimen Hospital, Beijing University of Chinese Medicine between December 2020 and February 2022, were selected based on the following criteria:

#### 2.1.1 Inclusion criteria

①Patients meeting the diagnostic criteria for diabetic foot; ②Grade 2 according to the Infections Diseases Society of America (IDSA) (Lavery et al., 2007); ③The wound surface was confirmed to be infected by pathogenic microorganisms through culture; ④Glycated hemoglobin ≤8%; ⑤Ages between 18 and 85 years, regardless of gender; ⑥Ankle-brachial index (ABI) ≥ 0.4; ⑦The wound area was within the range of 1 cm × 1 cm–10 cm × 10 cm (If the subjects have ≥2 wounds, the largest one would be considered as the study object); ⑧Patients voluntarily participated in this trial and signed informed consent form.

#### 2.1.2 Exclusion criteria

①Patients who were allergic to FFHB or ACAWD; ②Those who used antibiotics in anyway within 1 week before treatment; ③Severe heart, liver and renal insufficiency that seriously affected the safety and treatment of subjects were ruled out by the investigator; ④Patients with foot ulcer caused by venous, neoplastic, radioactive, simple arterial and other non-diabetic reasons; ⑤Serum albumin <25 g/L; ⑥Hemoglobin <90 g/L; ⑦ Platelet is lower than the lower limit of normal value; ⑧Those who have pregnancy or family planning, or pregnant or lactating women; ⑨Patients with cognitive dysfunction who cannot give full informed consent; ⑩At the discretion of the investigator, the patient was unable to complete the study or to comply with the requirements of the study.

#### 2.1.3 Elimination criteria

①During the trial, the inclusion/exclusion criteria were violated; ②During the experiment, subjects applied drugs or dressings to the affected area that were explicitly identified as having antibacterial properties in their instructions or product literature; ③The subjects received vascular intervention during the experiment.

### 2.2 Interventions

#### 2.2.1 Treatment method

Subjects were divided into control and experimental groups through minimization randomization.

Both groups were administered a systematic basic medical treatment regimen tailored to individual patient needs. This included a diabetic diet and medications aimed at controlling blood pressure, blood glucose, and lipid levels. It should be noted that the specific medications varied among patients due to the presence of multiple comorbidities and long-term prescriptions. While it is infeasible to list all drugs, they encompassed a broad range of commonly used antihypertensives, antidiabetics, and lipid-lowering agents.

In addition to the basic treatment, FFHB (Shandong Hanfang Pharmaceutical Co., Ltd., commercially available) was used in the treatment group. After treatment, the wound was conventionally wrapped. And the dressing was changed once a day, for a total of 2 weeks. In the control group, in addition to the basic treatment, ACAWD (Lomanos (China) Medical Products Co., Ltd., commercially available) was applied to the wound surface for 2 weeks and then conventionally wrapped. And the dressing was changed once a day. Since the two drugs studied are for external use, professional doctors are required to change the dressing. In the process, they can distinguish the differences. Therefore, this trial is an open clinical study. The wound dressing change methods are detailed in the Supplementary Material S1.

#### 2.2.2 Preparation of FFHB

FFHB is produced by Shandong Hanfang Pharmaceutical Co., Ltd. It contains Forsythiae Fructus 80 g, Phellodendri Chinensis Cortex 40 g, Lonicerae Japonicae Flos 40 g, Taraxaci Herba 40 g, and Scolopendra 2.4 g (Chinese Pharmacopoeia Commission, 2015).

Procedure: Decoct the above ingredients with water for three times,1 h for the first time, 45 min for the second, 30 min for the third time, combine the decoctions, filter, and concentrate the filtrates to a thin extract with a relative density of 1.10–1.15 (50°C), add ethanol and adjust the concentration of ethanol to 70%, stand for 24 h and filter, recover ethanol to no ethanolic smell in vacuum, add water to 1,000 mL, stir well, store at a low temperature for 24 h, filter, pack and sterilize. The chemical analysis follows the standards established by the ConPhyMP statement (Heinrich et al., 2022). The identification methods are detailed in the Supplementary Material S2.

### 2.3 Follow-up plan

**FIGURE 1:**
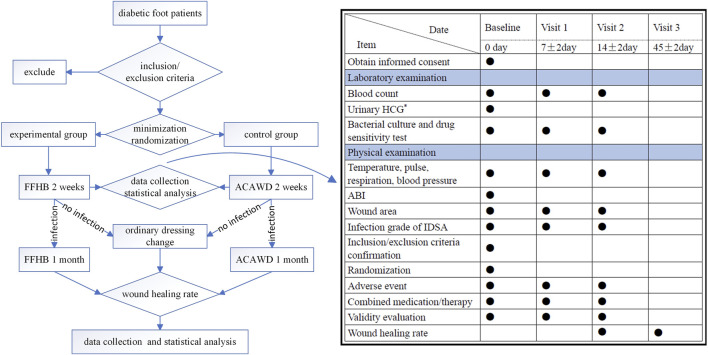
Flowchart ●: Required item; *: When joining the group, women of childbearing age are examined; ABI: Ankle-brachial index; HCG, human chorionic gonadotropin; IDSA, Infections Diseases Society of America.

All patients were discharged after 2 weeks of treatment. If the patient still has an infection, they should continue to use drug anti-infection treatment. Continue to use FFHB or ACAWD, respectively. If the infection has been eliminated, it will be changed to ordinary dressing change treatment. Use saline cotton ball to clean the wound, and cover a proper amount of sterile gauze on it. Then, bandage it with gauze bandage. One month later, the patients in the two groups were followed up by outpatient service or social software, and whether the wounds healed or not was counted, see flowchart for details (Figure 1).

### 2.4 Observation indicators

#### 2.4.1 Baseline data collection

Before entering the group, demographic data (age and gender) of subjects in both groups, concomitant diseases, bacterial type of infection (Gram staining), ABI and DF ulcer history and ulcer site were collected as baseline data. Efficacy and safety indicators were also assessed for all subjects at baseline (enrollment), at Visit 1 (7 ± 2 days dosing), and at Visit 2 (14 ± 2 days dosing).

#### 2.4.2 Primary efficacy measurements

①Pathogenic microorganism culture: using sterile cotton swab by Levin’s method to collect wound secretion of patients, conducting a bacterial culture and drug sensitivity test to observe whether pathogenic microorganisms on the wound surface were eradicated following drug administration;
②Minimum inhibitory concentration (MIC) value: The susceptibility results of cultures were used to detect whether the MIC of pathogenic microorganisms against different antibacterial drugs was changed.
③Infection control rate: The rate of the IDSA grade of DFI patients decreasing from grade 2 to grade 1. The judgment results were subjected to blind evaluation by clinical experts. If the judgment results are inconsistent, the researcher shall make a new judgment and make an explanation.

#### 2.4.3 Secondary efficacy measurements

①Wound surface area and wound area healing rate: The three-dimensional wound measurement and recording device of eKare inSightTM was used to measure the wound surface area, so that the camera was perpendicular to the wound surface. The deflection angle was less than 15° and the distance to the wound surface was about 40 ± 5 cm, ensuring that the wound surface was located in the center of the display screen. When the device was automatically recognized as 3D mode, the wound surface and the device were kept stationary for photographing. Wound area healing rate = [(baseline wound area—visit wound area)/baseline wound area] × 100%; The wound areas were recorded twice by two researchers and the average value was taken.
②Cost-effect ratio: The direct medical costs (bed cost, nursing cost, consumables cost, drug replacement cost and medication cost) of the two treatment methods were calculated and divided by the negative rate of microbial culture, infection control rate and wound area healing rate, respectively. The economies of the FFHB and ACAWD were compared by comparing the costs required to obtain the 1% negative rate of microbial culture, 1% infection control rate and 1% wound area healing rate.
③Wound healing rate: One month after the subjects were discharged from the group, the wound healing of the subjects in the two groups were followed up, and the two researchers made a judgment on whether they healed or not. If there are differences between the two researchers, they can reach an agreement through discussion, or ask the third researcher to make a ruling.

#### 2.4.4 Safety assessments

All adverse events observed during the treatment, including any symptoms and signs, were considered as safety indicators:

1) Drug Allergy: If patients exhibit allergic reactions such as skin itching, papules, erythema, wheals, eczema, or blisters during treatment, they should discontinue treatment immediately. Mild reactions may resolve spontaneously, whereas severe reactions should be managed with antiallergic treatments under medical guidance.
2) Wound Bleeding: In the event of bleeding, immediate compression should be applied to the wound. If the bleeding ceases, treatment may proceed as planned. Should the bleeding persist, the experiment should be halted, and surgical intervention may be necessary to stop the bleeding.
3) Exacerbation of Infection: If there’s an escalation in infection, the experiment should be stopped immediately. Affected patients should receive intravenous administration of appropriate antibiotics.

### 2.5 Allocation

The centralized MagMinDA clinical trial randomization system was utilized for participant enrollment. Stratification factors were determined based on patients’ baseline characteristics and factors influencing infection control, such as the bacterial type of infection (determined by Gram staining as G-/G+) and the ABI value (either ≤0.7 or >0.7). Using the minimization algorithm principle, the allocation probability for each patient was calculated. When the first subject was completely randomized, from the second study object, the difference of prognostic factors between the two groups was calculated after the study object was divided into specific groups. According to the principle of minimizing the difference, the research objects were randomly grouped according to the distribution probability. This approach ensured that patients were assigned to the most appropriate treatment group, guaranteeing a balanced distribution of control factors between the groups.

### 2.6 Statistical method

Experimental data was processed using SPSS 21.0, with measurement data represented as mean ± standard deviation ( $\bar{x}$ ±s). The normal distribution test and variance homogeneity test were performed. If the conditions were met, *t*-test was used. For skewed distribution data that did not meet the conditions, the Kruskal–Wallis Test was performed, expressing the data as median [upper and lower quartiles) (M(P25,P75)]. The count data were subjected to chi-square test. A *p*-value of <0.05 was considered statistically significant.

## 3 Results

### 3.1 Comparison of basic conditions of subjects between the two groups when they were enrolled

A total of 42 subjects were included, including 22 in the experimental group and 20 in the control group. All the subjects obtained informed consent. Two patients from the experimental group dropped out due to the COVID-19 outbreak and their failure to strictly follow the dressing change protocol. There were 42 cases in the FAS set and 40 in the PPS set. The FAS set was used for baseline data analysis. The two subjects’ dropout was due to completely random deletions, and the deletion rate was low. Thus, the PPS set was employed for follow-up data analysis.

Between the two groups, there were no significant differences in demographic data (age, gender), concomitant diseases, bacterial type of infection (based on Gram staining), ABI, DF ulcer history, or ulcer site (*p* > 0.05) (Table 1).

**TABLE 1:** Basic conditions of subjects in the two groups when entering the group.

Index	Experimental group	Control group	Statistic	*p*-value
Age (mean ± sd)	66.55 ± 11.959	61.55 ± 10.283	t = 1.444	0.156
Gender (male/female, case)	15/7	14/6	χ^2^ = 0.018	0.899
Renal insufficiency (none/yes, case)	21/1	19/1	-	1.000
Diabetic peripheral neuropathy (none/yes, case)	6/16	2/18	χ^2^ = 2.027	0.155
Hypoproteinemia (none/yes, case)	13/9	16/4	χ^2^ = 2.143	0.143
Hypertension (none/yes, case)	9/13	8/12	χ^2^ = 0.004	0.952
Hyperlipidemia (none/yes, Cases)	13/9	10/10	χ^2^ = 0.349	0.554
Gram staining (G-/G+, case)	17/5	16/4	χ^2^ = 0.046	0.830
ABI (>0.7/≤0.7, case)	8/14	10/10	χ^2^ = 0.795	0.372
History of amputation (none/yes, Cases)	11/11	9/11	χ^2^ = 0.105	0.746
Ulcer site (left foot/right foot, case)	14/8	12/8	χ^2^ = 0.059	0.808
Ulcer site (forefoot/midfoot/hindfoot, case)	13/8/1	11/8/1	χ^2^ = 0.353	1.000

### 3.2 Comparison of pathogenic microorganisms and infection control between the two groups

The culture report of pathogenic microorganisms from the subjects was analyzed. If no bacteria were cultured at Visit 2, compared to the baseline data, it was determined as “Yes”; otherwise, it was marked as “NO.” The probability of negative wound culture was 30% in the experimental group and 10% in the control group. There was no significant difference between the two groups (χ2 = 1.406, *p* = 0.236) (Table 2).

**TABLE 2:** Pathogenic microorganisms and infection control of subjects in two groups.

Index	Experimental group	Control group	Statistic	*p*-value
Culture without bacteria (yes/no, case)	6/14	2/18	χ^2^ = 1.406	0.236
MIC value (increase/decrease, case)	17/15	25/26	χ^2^ = 0.133	0.716
Infection downgraded to IDSA1 (yes/no, case)	12/8	5/15	χ^2^ = 5.013	0.025

The MIC values reported in all pathogenic microorganism drug sensitivity reports during the subject visit period were analyzed. If a change in MIC was observed, it was considered a positive event. There were a total of 32 positive events in the test group and 51 in the control group were obtained. MIC was determined to be increased if it was higher than its previous value, and was noted to be decreased if it was lower than its previous value. There was no significant difference between the two groups (χ2 = 0.133, *p* = 0.716) (Table 2).

Relevant data, including wound photos and subject symptoms, were collected to ascertain whether the infection grade of the patients decreased from IDSA2 to 1. The infection control rate for the test group at visit 2 was 60%, while it was 25% in the control group. The difference between the two groups was statistically significant (χ2 = 5.013, *p* = 0.025), and the effect in the experimental group was superior to that in the control group (Table 2).

### 3.3 Analysis of wound surface area and wound area healing rate of subjects in two groups

The overall wound area decreased in both groups of subjects. In the intra-group comparison, there was a significant difference between Visit 1 and baseline (*t* = 2.437, *p* = 0.025) and between Visit 2 and baseline in the experimental group (*t* = 3.539, *p* = 0.002). However, there was no statistical difference between Visit 1 and baseline in the control group (*t* = 1.421, *p* = 0.177), and there was a statistical difference between Visit 2 and baseline (*t* = 3.012, *p* = 0.007) (Table 3).

**TABLE 3:** Comparison of wound surface areas between the two groups within the same group.

Group	Baseline (cm^2^)	Visit 1 (cm^2^)	Visit 2 (cm^2^)	*p*-value (baseline-visit 1)	*p*-value (baseline-visit 2)
Experimental group	12.43 ± 9.07	10.32 ± 6.61	8.09 ± 6.39	0.025	0.002
Control group	12.49 ± 11.10	10.83 ± 9.75	8.68 ± 7.49	0.177	0.007

The wound area healing rates of the experimental group and the control group were calculated separately. For both Visit 1 and Visit 2, there was no significant difference between the two groups (*p* > 0.05). The median wound area healing rate in the Visit 2 experimental group was 34.4%, while that in the control group was 33.3% (Table 4).

**TABLE 4:** Wound area healing rates of subjects in the two groups.

Date	Wound healing rate	Experimental group	Control group	Statistic	*p*-value
Visit 1	N (missing)	19 (1)	15 (5)	χ^2^ = −0.884	0.376
	M(P25,P75) (%)	13.6 (-26.7,27.0)	19.0 (1.0,34.8)		
Visit 2	N (missing)	20 (0)	19 (1)	χ^2^ = −0.337	0.736
	M(P25,P75) (%)	34.4 (15.0,56.1)	33.3 (19.7,52.2)		

### 3.4 Cost-effectiveness analysis of two groups of subjects

Due to limitations in data accuracy, this study only calculated the main direct medical costs for treating DFI wounds in subjects, including bed fee (normal), nursing fee (level II), consumables fee (gauze and cotton balls), drug replacement fee (incurred during the process of dressing change), and drug cost (the unit price of FFHB is 39.2 yuan, and that of ACAWD is 340 yuan). The total cost for 14 days was 1971.9 yuan in the experimental group and 6173.86 yuan in the control group (Table 5).

**TABLE 5:** Direct medical costs for subjects in two groups.

Cost items	Experimental group		Control group	
	Unit price (yuan)	Quantity×Days	Unit price (yuan)	Quantity×Days
Bed fee	50	1 × 14	50	1 × 14
Nursing expenses	26	1 × 14	26	1 × 14
Consumables Fees	0.33	5 × 14	0.33	3 × 14
Exchange medicine fee	24	1 × 14	24	1 × 14
Expenses for medicine	39.2	1 × 14	34 0	1 × 14
Total	1971.9		6,173.86	

From the previous analysis, the probability of negative wound culture was 30% in the experimental group and 10% in the control group. The infection control rate of the experimental group was 60% and that of the control group was 25%. The median wound area healing rate was 34.4% in the experimental group and 33.3% in the control group. Through calculation, we respectively obtained the costs required to achieve 1% negative rate of microbial culture, 1% infection control rate and 1% wound area healing rate in the two groups, with the results kept to two decimal places. The cost to achieve a 1% negative rate of microbial culture, 1% infection control rate, and 1% wound area healing rate in the experimental group were 65.73, 32.87 and 57.32 yuan, respectively. The control group was 617.39, 246.95 and 185.40 yuan, respectively. The experimental group had more pharmacoeconomic advantages than the control group.

### 3.5 Wound healing rate of two groups of subjects

After 1 month follow-up, all 20 cases in the experimental group were healed (with a healing rate of 100%), while 15 cases in the control group were healed (the healing rate was 75%). The difference between the two groups was statistically significant (*p* = 0.047) (Table 6).

**TABLE 6:** Wound healing of two groups of subjects.

Index	Experimental group	Control group	*p*-value
Wound healed (yes/none, case)	20/0	15/5	0.047

## 4 Adverse event conditions in both groups

No significant adverse reactions were reported in either group during the trial.

## 5 Discussion

### 5.1 Understanding diabetic foot infections (DFI)

In 2019, the International Working Group on Diabetic Foot defined DF as an infection, ulcer or tissue damage in the foot of a patient who was newly diagnosed with diabetes or had a history of diabetes, usually accompanied by lower extremity neuropathy and/or peripheral artery disease, and defined DFI as a clinical manifestation of inflammation in tissues below the ankle in diabetic patients (Bus et al., 2020). DFI is an important factor for the development and deterioration of DF, which consuming vast medical resources, including anti-infection treatment and surgery (Lavery et al., 2006; Hao et al., 2014; Lazzarini et al., 2018). A large portion of DFI wounds fail to heal, which is related to the infection (including osteomyelitis) and/or gangrene development of the foot or lower limb and the increased risk of lower limb amputation (Pecoraro et al., 1990; Adler et al., 1999). Effective and timely treatment of DFI is crucial for promoting wound healing and saving patients’ limbs and lives.

### 5.2 Comparing efficacies of two treatment modalities

In this study, the experimental group used the hydropathic compress method with FFHB coated on medical gauze. In contrast, the control group employed ACAWD combined with silver ion and calcium alginate. Both treatment exhibited antibacterial effects. After absorbing the exudate, ACAWD was similar to FFHB in, maintaining a moist local wound surface. The morphology and mechanism of action of the selected treatments were similar in both groups. Numerous clinical studies have proved that silver ion dressings have a positive effect on DFI (Yang et al., 2021; Luo et al., 2022). In this study, there was no statistical significance between the negative rate of wound culture in the experimental group and that in the control group, proving that for DFI patients with IDSA2 grade, the bactericidal effect of FFHB was comparable to that of ACAWD.

### 5.3 Exploring FFHB’s antibacterial properties and mechanisms

Modern pharmacological studies have demonstrated that FFHB can inhibit *S. aureus*, *Pseudomonas aeruginosa* and *Proteus* (Sun et al., 2020). The extracts of Taraxacum mongolicum and Honeysuckle Flower showed strong inhibitory activity against *Proteus*. The centipede medicinal extract has strong inhibitory activity on *P. aeruginosa*, *S. aureus*, *Proteus*, and *Klebsiella pneumoniae* (Sun et al., 2020). The above basic studies confirmed that FFHB had a positive antibacterial effect, as evidenced in our clinical trials.

MIC refers to the minimum concentration of antibacterial drugs to inhibit the growth of a certain microorganism. Unlike conventional drug sensitivity tests that detect at a single concentration, MIC can quantitatively reflect the drug resistance of pathogenic microorganisms. Therefore, this index is often used for monitoring the drug resistance of pathogenic microorganisms and further guiding clinical medication. If the MIC value of an antibacterial agent against a pathogenic microorganism increase, it is considered that a MIC shift of the antibacterial agent has occurred. If the MIC value reaches a certain limit, it may cause the treatment failure of the antibacterial agent, and exert pressure on clinical treatment of DFI. Studies have shown that antibacterial drugs below the MIC not only enhance the hemolytic activity of *S. aureus* (Kuroda et al., 2007) but also promote the expression of virulence factors (Shang et al., 2019), but also stimulate the local formation of biofilm (Jin et al., 2020), lead to induce the production of drug-resistant bacteria (Bhattacharya et al., 2017). There was no statistical difference between the two groups, indicating no significant variance between the two drugs in regulating drug resistance of pathogenic microorganisms in DFI infections.

### 5.4 Clinical improvements and underlying pharmacological mechanisms

If the IDSA is downgraded from level 2 to level 1, it indicates successful for anti-infective therapy. The infection control rate of the experimental group in Visit 2 was 60% and that of the control group was 25%, and the effect of the experimental group was better than that of the control group. This is probably related to the multi-metabolite and multi-target property of FFHB. Its pharmacological effects may not only lie in the direct killing of pathogenic microorganisms, but also be related to the regulation of wound cell molecular biological characteristics, resistance to the formation of bacterial biofilm, and improvement of the wound exudate microenvironment. Further basic research and clinical trial verification are needed to elaborate on the mechanism of FFHB in the treatment of DFI.

The intra-group comparison of wound area at Visit 2 was statistically significant compared with baseline, indicating that the wounds of the subjects in the two groups generally showed a healing trend. Differences for the treatment group at Visit 1 compared to baseline were statistically significant, whereas those for the control group were not. This might be related to the toxicity of silver ions to normal tissues, which slowed the wound healing. Previous research demonstrated that nano-silver solution could inhibit fibroblasts cultured *in vitro* (FU et al., 2010).

The end point of treatment of diabetic foot should be wound healing, and patients can return to family and society. Infection control is an important step in wound healing. With good antibacterial effect and relatively weak tissue toxicity of FFHB, the wound healing rate of the experimental group was significantly better than that of the control group after 1 month of follow-up.

The basic research of FFHB has shown that it can promote wound healing by inhibiting bacterial reproduction, reducing local inflammation and edema, maintaining a wet healing environment, and promoting wound granulation growth (Zhang et al., 2020; Zheng et al., 2021). Clinical studies have also shown that FFHB can inhibit the body’s synthesis of advanced glycation end products to reduce inflammation, remove wound pathogenic microorganisms, control and prevent local infection, promote autolysis of necrotic tissue, and increase the number of growth factors to promote wound healing in diabetic foot (You-shan and Bo-hua, 2014; Wang et al., 2019).

### 5.5 Economic evaluation: Cost-effectiveness of FFHB vs. ACAWD

Cost-effectiveness analysis aims to identify the most economical treatment plan to achieve a desired treatment outcome. The cost to effect ratio is expressed as the cost required to achieve a unit effect. The cost of pharmacoeconomics includes direct cost, indirect cost and negative cost. Direct costs include both direct medical cost and non-medical expenses. Limited by the precise availability of data, many pharmacoeconomic estimates calculate only direct medical costs. It has been calculated in this study that the cost of the control group was significantly higher than that of the experimental group. From an economic viewpoint, FFHB offered a notable price advantage over ACAWD. In combination with the lack of obvious adverse reactions in both groups of subjects, FFHB was a safe and economical medication for DF mild infection.

### 5.6 Limitations and future directions

The subgroup analysis of some test indicators, such as MIC trend change for a specific pathogenic microorganism, revealed that more clinical data is necessary to achieve a substantial sample size, and we should expand sample size in future studies. Second, the observation and follow-up time in this trial was relatively short. The wound healing time of most DFI patients was often several months, and the 14-day follow-up time was relatively short. Further prolongation of the observation period is required to demonstrate the long-term efficacy of the test drug. In order to collect complete data on subjects, all subjects included in this study were hospitalized. Outpatients may be considered for inclusion in further studies to make the results more consistent with the real-world situation. Finally, since this study is a clinical trial, there is a lack of verification for the comparison of the *in vitro* antibacterial effects of FFHB and ACAWD.

## 6 Conclusion

This study compared the effects of FFHB and ACAWD in the treatment of DFI. The results demonstrated that FFHB has significant advantages in promoting wound healing, inhibiting bacterial proliferation, reducing local inflammation and edema, among others. Moreover, when compared to ACAWD, FFHB showed greater cost-effectiveness, offering a safe and economical medicinal choice for mild DF infections.

The study also points out some limitations, such as sample size and follow-up duration, which might influence the conclusions. However, overall, this research provides robust evidence for the application of FFHB in the treatment of DFI and offers valuable guidance for further clinical research and practice.

## Data Availability

The raw data supporting the conclusion of this article will be made available by the authors, without undue reservation.

## Abbreviations

ABI, Ankle brachial index; ACAWD, Antimicrobial Calcium Alginate Wound Dressing; DF, Diabetic foot; DFI, Diabetic foot infection; FAS, Full analysis set; FFHB, Fufang Huangbai Fluid; G-, Gram negative bacteria; G+, Gram positive bacteria; IDSA, Infectious Diseases Society of America; MIC, Minimum inhibitory concentration; PPS, Per protocol set.
