# Supplementary material for: Efficacy and pharmacoeconomic advantages of Fufang Huangbai Fluid hydropathic compress in diabetic foot infections: a comparative clinical study with antimicrobial calcium alginate wound dressing
Source: Front Pharmacol. 2024 Jan 22;15:1285946. doi: 10.3389/fphar.2024.1285946 (PMC10839075; doi:10.3389/fphar.2024.1285946)
Supplement: Supplementary file 1 [file Table1.DOCX]

**Operation flow of wound debridement and dressing change**

1. Remove the outer dressing: Remove the outer bandage and dressing with your hands and remove the dressing close to the wound with tweezers. The direction of the dressing is parallel to the long axis of the wound. If the inner dressing adheres to the wound, wet application of sterile saline should be used and then slowly removed.
2. Observe the wound carefully: Look at the secretion, carrion and granulation tissue of the wound.
3. Clean and treat the wound: Hold a pair of sterile tweezers to pass the saline cotton ball in the medicine bowl to the other hand, and use saline cotton ball to scrub the secretion and carrion on the wound (can be combined with the specific conditions of the wound, sharp debridement of necrotic tissue or carrion, but drugs or dressings with clear antibacterial effects such as iodophor or hydrogen peroxide should not be used).
4. Observe the wound again: Carefully observe the granulation tissue and wound area of the wound.

5.

1. Experimental group (FFHB): Soak the original liquid of FFHB into 10 layers (the thinnest layer of gauze) sterile gauze (with no dripping). Cover the wound with sterile gauze, 2cm beyond the edge of the wound. If the wound is deep, use sterile tweezers to insert the soaked sterile gauze into the wound. The wound was soaked for 10 minutes and then removed and discarded. Moreover, continue to cover the wound with sterile gauze soaked with FFHB as described above. At the same time, pick up a proper amount of sterile gauze (to avoid obvious penetration of FFHB) and cover it on the soaked sterile gauze, and bandage it with gauze bandage.
2. Control group (ACAWD): Take one piece of ACAWD, use sterile tissue scissors, cut the edges of large and small wounds by 2cm, and cover the wound surface. If the wound is deep, the soaked sterile gauze should be inserted into the wound with sterile tweezers. At the same time, pick up a proper amount of sterile gauze and cover it on the ACAWD, and bandage it with gauze bandage.

6. Clean up the dressing items and assist patients in sorting out their clothes.
