# Supplementary material for: Efficacy and pharmacoeconomic advantages of Fufang Huangbai Fluid hydropathic compress in diabetic foot infections: a comparative clinical study with antimicrobial calcium alginate wound dressing
Source: Front Pharmacol. 2024 Jan 22;15:1285946. doi: 10.3389/fphar.2024.1285946 (PMC10839075; doi:10.3389/fphar.2024.1285946)
Supplement: Supplementary file 2 [file Table2.DOCX]

**Thin Layer Chromatography (TLC) Identification Methods for Fufang Huangbai Fluid (FFHB)**

Source：Committee, N.P. (2015). *Pharmacopoeia of the People’s Republic of China.* Beijing: The Medicine Science and Technology Press of China.

(1) To 40 ml, extract by shaking with two 40-ml quantities of n-butanol saturated with water, combine the *n*-butanol extracts, wash with 40 ml of ammonia *TS*, separate the n-butanol layer and evaporate to dryness, dissolve the residue in 1 ml of methanol as the test solution. Decoct 1 g of Forsythiae Fructus reference drug with 40 ml of water for 30 minutes, filter, prepare the reference drug solution in the same manner as described under the test solution. Dissolve forsythin CRS in methanol to produce a solution containing 1 mg per ml as the reference solution. Carry out the method for thin layer chromatography (0502) using silica gel G as the coating substance and the mixture of chloroform, acetone, methanol and formic acid (12: 2.5: 2: 0.2) as the mobile phase. Apply separately 5 μl of the above three solutions to the plate. After developing and removal of the plate, dry in air. Spray with 10%sulfuric acid in ethanol, heat at 105℃ to the spots clear and examine in daylight. The spots in the chromatogram obtained with the test solution correspond in position and colour to the spots in the chromatogram obtained with the reference drug solution and the reference solution.

(2)To 20 ml, add hydrochloric acid and adjust the pH value to 2. Extract the solution with two 20-ml quantities of chloroform, combine the extracts, evaporate to dryness, dissolve the residue in 1 ml of methanol as the test solution. To 0.1g of Phellodendri Chinensis Cortex reference drug, add 5 ml of ethanol, heat under reflux for 15 minutes, filter and use the filtrate as the reference drug solution. Dissolve berberine hydrochloride CRS in ethanol to produce a solution containing 0.1 mg per ml as the reference solution. Carry out the method for thin layer chromatography (0502), using silica gel G as the coating substance and the mixture of *n*-butanol, glacial acetic acid and water (7 : 1 : 2) as the mobile phase. Apply separately 2 μl of the above three solutions to the plate. After developing and removal of the plate, dry in air. Examine under ultraviolet light at 365 nm. The fluorescent spots in the chromatogram obtained with the test solution correspond in position and colour to the spots in the chromatogram obtained with the reference drug solution and the reference solution.

(3)To 40 ml, extract by shaking with two 40-ml quantities of ethyl acetate, combine the ethyl acetate extracts, evaporate to dryness, and dissolve the residue in 1 ml of methanol as the test solution. Decoct 1 g of each of Lonicerae Japonicae Flos reference drug and Taraxaci Herba reference drug with 40 ml of water for 30 minutes separately, prepare the reference drug solution in the same manner as described under the test solution.Carry out the method for thin layer chromatography (0502), using silica gel G as the coating substance and the upper layer of a mixture of butyl acetate, formic acid and water (14 : 5 : 5) as the mobile phase. Apply separately 5 ul of the above three solutions to the plate. After developing and removal of the plate, dry in air. Examine under ultraviolet light at 365 nm. The fluorescent spots in the chromatogram obtained with the test solution correspond in position and colour to the spots in the chromatogram obtained with the reference drug solution.

It contains not less than 60 μg of forsythin (C_27_H_34_O_11_) per ml, referred to Forsythiae Fructus, and contains not less than 10.0 ug of berberine hydrochloride (C_20_H_17_NO_4_・HCl)per ml, referred to Phellodendri Chinensis Cortex.
